# Pityriasis rubra pilaris nach COVID-19-Impfung: Kausaler Zusammenhang oder Koinzidenz?

**DOI:** 10.1007/s00105-022-04972-z

**Published:** 2022-03-16

**Authors:** A. C. Bramhoff, U. Wesselmann, S. T. Bender, A. V. Berghoff, S. C. Hofmann, G. Balakirski

**Affiliations:** Zentrum für Dermatologie, Allergologie und Dermatochirurgie, Helios Universitätsklinikum Wuppertal, Universität Witten/Herdecke, Heusnerstr. 40, 42283 Wuppertal, Deutschland

**Keywords:** COVID-19-Impfstoff, mRNA-Impfstoff, Kutane Impfreaktion, Pityriasis rubra pilaris, Systemische Retinoide, COVID-19 vaccine, mRNA vaccine, Vaccination reaction, Pityriasis rubra pilaris, Systemic retinoids

## Abstract

Seit der Markteinführung der COVID-19-Impfstoffe werden regelmäßig kutane Nebenwirkungen dieser Vakzine beschrieben. Diese beinhalten unter anderem lokale Impfreaktionen (insbesondere den sog. COVID-Arm), urtikarielle, makulopapulöse und pityriasiforme Exantheme oder vorübergehende Exazerbationen einer vorbestehenden chronisch entzündlichen Hauterkrankung. Wir berichten über 3 Fälle einer Pityriasis rubra pilaris, die in engem zeitlichen Zusammenhang mit der Verabreichung eines COVID-19-Impfstoffs erstmals aufgetreten sind.

## Abstract (en)

Numerous cutaneous side effects associated with COVID-19 vaccines have been described since their clinical approval. These include, among others, injection site reactions, urticarial, maculopapular and pityriasiform rashes or temporary exacerbations of a pre-existing chronic inflammatory skin disease. Herein we report about three cases of pityriasis rubra pilaris that occurred for the first time in close temporal relationship with the administration of a COVID-19 vaccine.

Die durch das neue SARS-CoV-2-Virus verursachte COVID-19-Pandemie hat seit Anfang 2020 das Leben der Menschen in vielen Bereichen verändert. Um der Pandemie entgegenzuwirken, wurden Anfang 2021 weltweit Impfkampagnen gestartet. Dabei wurden zum ersten Mal mRNA-Vakzine zugelassen und eingesetzt [1], im Verlauf auch Vektorimpfstoffe. Die bisher beschriebenen kutanen Nebenwirkungen der Impfstoffe beinhalten unter anderem lokale Impfreaktionen (insbesondere den sog. COVID-Arm), urtikarielle, makulopapulöse und pityriasiforme Exantheme oder vorübergehende Exazerbationen einer vorbestehenden chronisch entzündlichen Hauterkrankung wie beispielsweise eines Lupus erythematodes [2–5].

Wir berichten nun über 3 Fälle einer Pityriasis rubra pilaris (PRP), die in engem zeitlichen Zusammenhang mit der Verabreichung eines COVID-19-Impfstoffs erstmals aufgetreten sind.

## Fall 1

**Figure 1:**
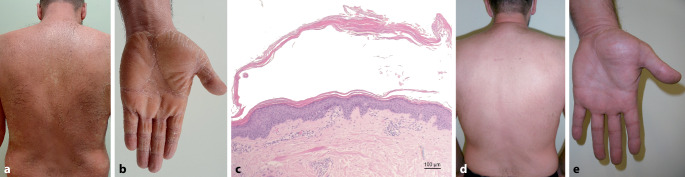

Der 50-jährige Patient stellte sich in unserer Klinik mit seit etwa 4 Wochen bestehenden stark juckenden Hautveränderungen an Stamm und Extremitäten sowie lachsfarbenen palmoplantaren Hyperkeratosen vor (Abb. 1a, b). Anamnestisch seien die ersten Hautläsionen etwa 9 Tage nach der Erstimpfung mit dem COVID-19-Vektorimpfstoff Vaxzevria® (AstraZeneca) aufgetreten. Chronische Erkrankungen, Allergien, jegliche Hauterkrankungen und Medikation in der Anamnese wurden verneint. Eine bereits auswärts begonnene Lokaltherapie mit einer Mometasonfuroat-haltigen Creme habe nicht zu einer Befundbesserung geführt. Eine PRP konnte histologisch bestätigt werden (Abb. 1c). Unter oraler Therapie mit Acitretin 35 mg/Tag sowie topischen Kortikosteroiden zeigte sich eine deutliche Regredienz des Hautbefundes. Die 2. COVID-19-Impfung erfolgte 12 Wochen nach der 1. Vakzination mit dem COVID-19-mRNA-Impfstoff Comirnaty® (BioNTech/Pfizer) und wurde problemlos vertragen. Zwölf Wochen nach Beginn der Systemtherapie zeigt sich die PRP aktuell gut kontrolliert (Abb. 1d, e) unter fortgeführter Therapie mit Acitretin.

## Fall 2

**Figure 2:**
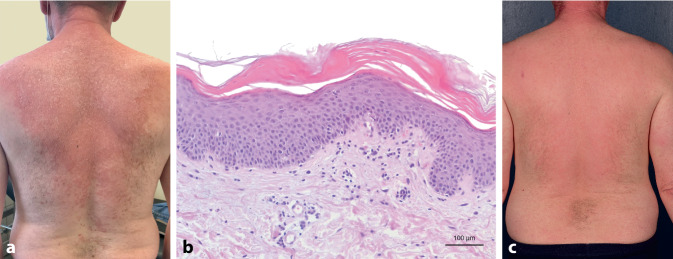

Der 58-jährige Patient stellte sich in unserer Klinik mit einem stark juckenden, schuppenden Erythem an Gesicht, Rumpf, Extremitäten sowie Palmae und Plantae vor (Abb. 2a), welches etwa 20 Tage nach Verabreichung der 2. Dosis des Impfstoffs Comirnaty® aufgetreten sei. Die bereits auswärts begonnene topische Therapie mit Prednicarbat-Creme und oraler Gabe von Prednisolon 30 mg/Tag habe keine Befundverbesserung erzielt. Der histologische Befund war vereinbar mit einer initialen PRP (Abb. 2b). Bis auf eine vorbekannte arterielle Hypertonie und einen Diabetes mellitus Typ I lagen keine chronischen Erkrankungen oder Allergien vor. Aufgrund der ausgeprägten palmoplantaren Beteiligung leiteten wir eine orale Therapie mit Alitretinoin 30 mg/Tag sowie eine topische Kortikosteroidtherapie ein. Sechs Wochen nach Therapiebeginn zeigt sich aktuell eine deutliche Besserung des Hautbefundes (Abb. 2c) bei nahezu vollständigem Sistieren des Pruritus.

## Fall 3

**Figure 3:**
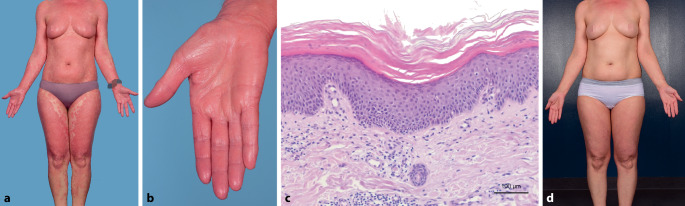

Die 60-jährige Patientin entwickelte stark juckende, konfluierende, teils schuppende, orangefarbene bis erythematöse Plaques am gesamten Integument (Abb. 3a, b) etwa 4 Wochen nach der Erstimpfung mit Vaxzevria®. Die ersten Läsionen seien an der Injektionsstelle am linken Oberarm aufgetreten mit nachfolgender Generalisation. Bis auf eine substituierte Hypothyreose und eine Rhinitis allergica bei Gräserpollenallergie lagen keine chronischen Erkrankungen vor. Bei ebenfalls fehlender Besserung unter einer topischen Therapie mit Mometasonfuroat wurden Probebiopsien entnommen, welche (Abb. 3c) in Zusammenschau mit der Klinik zur Diagnose einer PRP führten.

Da es unter einer initial eingeleiteten Therapie mit Acitretin 35 mg/Tag in Kombination mit topischen Kortikosteroiden nicht zur Befundbesserung kam, wurde nach 3 bis 4 Wochen eine Therapie mit Methotrexat 15 mg s.c. 1‑mal wöchentlich in Kombination mit 5 mg Folsäure am Folgetag ergänzt. Hierunter besserte sich der Hautbefund, sodass etwa 8 Wochen nach Therapiebeginn die Methotrexat(MTX)-Dosis auf 10 mg wöchentlich reduziert werden konnte (Abb. 3d, e).

In der Zwischenzeit erhielt die Patientin die zweite Dosis des gleichen COVID-19-Impfstoffs ohne merkliche Befundexazerbation.

PRP ist mit einer geschätzten Inzidenz von 1:50.000/Jahr eine seltene chronisch entzündliche Hauterkrankung, welche sich bevorzugt im Kindes- und Jugendalter sowie der 5. und 6. Lebensdekade manifestiert. Die Ätiologie ist unklar, wobei in einzelnen Fällen eine Triggerung durch Virusinfektionen (Hepatitis C, HIV), Impfungen oder Medikamente (u. a. Tyrosinkinase- und Checkpointinhibitoren), selten auch eine Assoziation mit Malignomen oder Hypothyreose beschrieben wurde [6, 7]. Einzelne Berichte einer infektassoziierten PRP nach COVID-19-Infektion liegen vor [8].

In der Literatur finden sich bisher nur 3 Fälle, die eine PRP-Erstmanifestation in zeitlicher Assoziation mit einer COVID-19-Impfung (in 2 Fällen nach Vaxzevria®, 1‑mal nach Comirnaty®) beschreiben [9–11]. In 2 Fällen traten die ersten Läsionen einige Tage, in dem dritten Fall 2 Wochen nach der ersten Impfdosis auf. Zwei Patienten hatten nach Reexposition mit dem gleichen Impfstoff eine Verschlechterung der PRP entwickelt. Die dritte Patientin erhielt aufgrund einer vorausgegangenen COVID-19-Infektion keine Zweitimpfung. Bei unseren 3 Patienten mit der klassisch-adulten Form der PRP traten erste PRP-Läsionen 9 Tage bis 4 Wochen nach der Verabreichung eines SARS-CoV-2-Impfstoffes auf. Nur 1 Patientin erhielt die Zweitimpfung mit der identischen Vakzine, 1 Patient wurde mit einem mRNA-Impfstoff geimpft bei vorheriger Impfung mit Vektorimpfstoff. Beide reexponierten Patienten erlitten keine Exazerbation der PRP.

Die PRP zählt ebenso wie die Psoriasis vulgaris zu den Th1-dominierten entzündlichen Dermatosen [12]. Da in Phase-1- und -2-Studien mit COVID-19-Impfstoff eine Aktivierung von Th1-Antworten nachgewiesen wurde [13] und die Häufung von 3 PRP-Erstdiagnosen innerhalb weniger Wochen an unserer Klinik ungewöhnlich ist, erscheint eine durch die Impfungen aktivierte Th1-Immunantwort als Trigger der PRP plausibel, möglicherweise auf dem Boden einer vorhandenen Prädisposition. Eine Befundbesserung konnte in allen bisher bekannten PRP-Fällen nach COVID-19-Impfung durch die üblichen Therapieschemata unter Einsatz von Retinoiden oder Immunsuppressiva wie MTX erzielt werden. Bei Therapieresistenz können auch Biologika (IL-17, IL-23 oder TNF-α-Antagonisten) eingesetzt werden [14]. Da Exazerbationen der PRP durch Reexposition mit COVID-19-Impfstoff vorkommen, sollte die Entscheidung für oder gegen eine Zweitimpfung individuell mit dem Patienten abgewogen werden.

## Fazit für die Praxis

- Durch die Aktivierung des Immunsystems durch die COVID-19-Impfstoffe kann es in seltenen Fällen bei entsprechender Prädisposition zum Erstauftreten chronisch entzündlicher Hauterkrankungen wie Pityriasis rubra pilaris kommen.
- In solchen Fällen stehen die Standardtherapieoptionen zur Verfügung.
- Da es durch die Verabreichung der nächsten Impfdosis zur Exazerbation der Hauterkrankung kommen kann, müssen die Patienten darüber gut aufgeklärt werden.
- Bei der gut kontrollierten Hauterkrankung können die weiteren Impfdosen dennoch komplikationsarm vertragen werden.
